# Can I trust this paper?

**DOI:** 10.3758/s13423-025-02740-3

**Published:** 2025-07-16

**Authors:** Andrey Anikin

**Affiliations:** Division of Cognitive Science, Department of Philosophy, Lund University, Box 192, SE-221 00 Lund, Sweden

**Keywords:** Research integrity, Replication, Statistics, Power

## Abstract

After a decade of data falsification scandals and replication failures in psychology and related empirical disciplines, there are urgent calls for open science and structural reform in the publishing industry. In the meantime, however, researchers need to learn how to recognize tell-tale signs of methodological and conceptual shortcomings that make a published claim suspect. I review four key problems and propose simple ways to detect them. First, the study may be fake; if in doubt, inspect the authors’ and journal’s profiles and request to see the raw data to check for inconsistencies. Second, there may be too little data; low precision of effect sizes is a clear warning sign of this. Third, the data may not be analyzed correctly; excessive flexibility in data analysis can be deduced from signs of data dredging and convoluted post hoc theorizing in the text, while violations of model assumptions can be detected by examining plots of observed data and model predictions. Fourth, the conclusions may not be justified by the data; common issues are inappropriate acceptance of the null hypothesis, biased meta-analyses, over-generalization over unmodeled variance, hidden confounds, and unspecific theoretical predictions. The main takeaways are to verify that the methodology is robust and to distinguish between what the actual results are and what the authors claim these results mean when citing empirical work. Critical evaluation of published evidence is an essential skill to develop as it can prevent researchers from pursuing unproductive avenues and ensure better trustworthiness of science as a whole.

## Introduction

I take notes when I read papers, often ending in an overall endorsement (*A very nice review of X, Cite as evidence of Y*) or a more critical assessment (*Not sure about their stats, Much ado about tiny effects,* or simply *Rubbish – ignore*). As the *rubbish* tags accumulate, I wonder how many questionable claims are still taken at face value, misguiding myself and other researchers. There is certainly no lack of cautionary tales about the replication crisis in both behavioral (Camerer et al., 2018; Nosek et al., 2022; Open Science Collaboration, 2015; Soto, 2019) and clinical (Begley & Ellis, 2012; Prinz et al., 2011) research, suboptimal statistical practices (Gelman & Loken, 2014; McShane et al., 2019), theory crisis in psychology (Muthukrishna & Henrich, 2019; Yarkoni, 2022), and poor research practices that undermine public trust in science (Bolland et al., 2025; Ioannidis, 2005; Ritchie, 2020). The proposed solutions focus on promoting open science, increasing the transparency of research and data analysis, sharing datasets, preregistering experiments, improving statistical practices, and implementing institutional reforms that would diminish the pressure to publish at all costs (Forstmeier et al., 2017; Gelman & Loken, 2014; Munafò et al., 2017; Nosek et al., 2018, 2019; Rubin, 2017; van den Akker et al., 2024). What seems to be missing in this literature, however, is a practical digest providing simple, yet comprehensive guidelines for a typical reader who may not be a statistician or a philosopher of science, but who nevertheless needs to know how to spot a problematic paper or a shaky claim.

To provide such a guide, however personal and simplified, is the ambition of this review. Its main subject is experimental research in psychology and related disciplines. Similar problems plague other fields of inquiry, but it is difficult to make recommendations so general that they would apply equally to literature in any field. The legitimacy of experimental evidence rests upon measuring a phenomenon accurately and drawing inferences based on these numbers. Numbers are reassuringly solid and objective, and yet quantitative evidence often remains inconsistent or inconclusive as researchers fail to agree and experiments fail to replicate. The root causes of these troubles range from problems with the evidence itself to questionable statistical practices and unwarranted theoretical conclusions. Each of these major concerns is briefly reviewed below, followed by concrete suggestions for how readers may detect them.

I would like to emphasize that these are merely rules of thumb, intended to be conceptually simple at the cost of over-simplifying some of the underlying issues. I do not offer a formal checklist or flowchart with standard steps and tests – instead, I aim to draw attention to several particularly serious or widespread problems with published claims and propose simple heuristics for detecting them, in the spirit of developing critical reading skills. Several formal guidelines and checklists have already been proposed for assessing research integrity, particularly in the context of clinical studies (Boughton et al., 2021; Grey et al., 2020; Mol et al., 2023; Parker et al., 2022), but these are too rigid for much psychological research. Besides, the focus in these tools is on detecting outright fraud, which is only a small part of evaluating the validity of published claims.

Inevitably, this paper reflects my personal experience and preferences that others may disagree with, both in terms of the highlighted problems and the suggested solutions (e.g., focusing on the uncertainty of effect sizes). This is not a comprehensive or systematic review of everything that can go wrong with a scientific publication. Despite these provisos, I see guidelines of this type as an essential complement to the gloomy assessments of the integrity and credibility of science presented as a structural problem (Bolland et al., 2025; Ritchie, 2020; Yarkoni, 2022). Upholding rigorous scientific standards is particularly urgent now that science is increasingly coming under attack politically. Raising awareness of the problem is commendable in itself, and addressing it fully will indeed require far-reaching institutional reforms, but in the meantime, papers are coming out in larger quantities than ever, and these papers are read and cited, often uncritically. Peer review, traditionally seen as a secure bulwark against shoddy research, is not robust enough to ensure high standards (Carneiro et al., 2020; Ioannidis, 2005; Ritchie, 2020; Smith, 2006; Tennant, 2018). Therefore, individual scientists need the tools and skills for evaluating the credibility of published evidence. There is hope that this is possible: people are good at guessing which studies will fail to replicate (Camerer et al., 2018), and large prediction markets yield replicability predictions with accuracy between 61% and 86% (Korbmacher et al., 2023). Thus, informed readers can learn when to be skeptical, mitigating the perverse selection pressure for bad science (Smaldino & McElreath, 2016) and contributing to the survival of good research. Let us therefore consider the four major categories of problems in turn.

## The data are not reliable

The worst-case scenario with empirical research is that the numbers themselves are suspect – not measured properly, falsified, or simply invented. Once considered an exception (Evans, 2001), outright data fraud is now known to occur in all disciplines. Only a few percent of researchers admit engaging in data fabrication or falsification, but the estimated prevalence is much higher (Fanelli, 2009; Heathers, 2025b; Williams & Roberts, 2016). A simple objective proxy for misconduct is the annual count of retracted papers because fraud is involved in between 20% (Grieneisen & Zhang, 2012) and 94% (Fang et al., 2013) of retractions. While about 4,500 papers were retracted in total from 1928 to 2011, their number increased nearly 20-fold from 2001 to 2010 (Grieneisen & Zhang, 2012), reaching over 4,000 retractions in 2022, over 10,000 in 2023 (Bolland et al., 2025), and about 5,000 in 2024 (https://retractionwatch.com/). This retraction boom far outstrips the growth in the total number of publications per year, which means that retracted reports represent an increasing share of all scientific output. This may be partly due to a pushback against scientific misconduct and the proliferation of paper mills, so this does not necessarily imply that your average researcher is increasingly corrupt (Mills et al., 2024; Petrou, 2024). On the other hand, even these tens of thousands of retractions are probably only the tip of the iceberg of fraudulent research (Bolland et al., 2025). Even worse, retracted papers often continue to circulate online and to be cited for years to come (Bolland et al., 2022), albeit at a reduced rate (Lu et al., 2013). Thus, there is a small, but non-negligible risk of running into outright fraud in the literature you read and cite. If you smell a rat, consider these four simple questions.

1. *Is the paper already retracted?*

This may seem obvious, but retracted publications continue to circulate online in many versions, often without a retraction notice (Bakker & Riegelman, 2018). Checking the official record on the journal’s webpage is a quick way to verify the retraction status and to find possible errata and corrigenda; another good resource is https://retractiondatabase.org. It is also worth checking whether there were any replication attempts (e.g., by browsing through “cited by” on Google Scholar), and whether they were successful.

(2) *Who wrote it?*

A large proportion of data fraud is committed by a few repeat offenders; Retraction Watch (https://retractionwatch.com/) lists five individuals with over 100 retracted papers each. Any paper by a repeat offender merits scrutiny. Another warning sign is unusually large research output in a short time, so it makes sense to check the authors’ previous publication record. Scientific seniority appears to be a poor predictor of misconduct: students and junior researchers (Gopalakrishna et al., 2022) are about as likely to be guilty as senior faculty members (Fang et al., 2013). For various reasons, the risk of scientific misconduct is highest in male researchers (Fang et al., 2013) working in competitive fields such as biomedicine, but many fields will boast one or two master fraudsters, and it is worth knowing them.

(3) *Where is it published?*

Predatory journals are proliferating, and in the absence of meaningful peer review or editorial oversight, data quality is anyone’s guess (Abdullah et al., 2024). Paper mills and fully or partially AI‐generated papers are also a growing concern (Abalkina & Bishop, 2023; Bolland et al., 2025). There are several lists of predatory journals to consult (Table 1), although the line between reputable and predatory journals is often blurred. However, well-established journals are not immune to the problem of fraud: in fact, there are *more* retractions and overblown claims in higher-ranked journals (Brembs, 2018; Brembs et al., 2013; Fang & Casadevall, 2011; Grieneisen & Zhang, 2012). Naturally, high-impact journals have more resources for investigating reported fraud and incentives for maintaining their reputation, but they also attract fraudsters because the potential rewards of cheating are so high, and the hunt for sensational findings may encourage lax editorial standards in the most competitive outlets (Ritchie, 2020; Serra-Garcia & Gneezy, 2021). Another ongoing change in the publishing landscape is the proliferation of preprints which are shared and cited before – or without – peer review and formal publication. Preprints enable rapid dissemination of novel findings (King, 2020), but some researchers are concerned that the lack of formal quality checks may translate into poor credibility (Da Silva, 2018; Van Schalkwyk et al., 2020). The evidence is still very limited, but there is actually no indication that the quality of reporting is noticeably lower in preprints than in regular articles (Carneiro et al., 2020; Nelson et al., 2022), and preprints can be withdrawn much more rapidly than publications in case a problem is discovered. If a preprint is changed, this is also likely to be documented, whereas undocumented “stealth corrections” of already published papers are surprisingly common (Aquarius et al., 2024). In sum, no journal or publishing format is fully safe, but openly predatory journals are the most likely habitats for fake papers.

(4) *Are raw observations available, and what do they look like?*

**Table 1:** A summary of critically evaluating published evidence and claims

Problem	Subtype	Definition	Warning signs	Suggested action	Key resources
Unreliable data	Fabricated	No data collection took place, entirely invented numbers	• Authors have multiple retractions • Published in a predatory journal • Data not available • If data available, check for reduced variation, repetitive values, flat distributions, lack of expected correlations between variables, perfectly balanced demographics in randomized groups, implausibly perfect results	Do not cite, contact authors, request to see data. If fraud confirmed: • Contact journal • Post concerns (e.g., https://blog.pubpeer.com/) • Contact the Committee on Publication Ethics (https://publicationethics.org/)	Retracted papers: https://retractionwatch.com Predatory journals: https://beallslist.net/https://cabells.com/https://kscien.org/https://earlywarning.fenqubiao.com/#/en/ (Bolland et al., 2025; Bordewijk et al., 2021)
	Falsified	Data modified to fit a hypothesis			
Not enough data	Low statistical power or precision of estimates	Empirical evidence is insufficient to meaningfully answer the research question	• Wide error bars or CIs relative to substantively important variation in effect size • Results highly sensitive to the chosen analysis • Unexpectedly large and exciting results, small sample size at any level (subject, item, etc.), opaque and complex analyses	Check for replications, cite with caution, expect real effect sizes to be smaller, ignore claims of no effect (high risk of false negatives)	(Brysbaert & Stevens, 2018; Westfall et al., 2014)
Data not analyzed properly	P-hacking = data dredging	Undisclosed flexibility in data collection and analysis	• No preregistration • No data analysis plan or scripts • More analyses performed than reported • Numerous tests with poor theoretical justification • Far-fetched hypotheses perfectly fit the results • Mismatch between study design and hypotheses	Cite with caution, high risk of false positives and poor generalizability (overfitting)	(Gelman & Loken, 2014; Kerr, 1998; Kruschke, 2021; Simmons et al., 2011)
	HARKing	Hypothesizing After the Results are Known			
	Poor model fit	Model does not adequately capture the data	• Underfitting: plots of raw data show unmodeled trends (nonlinear, heteroscedastic, autocorrelated, etc.) • Overfitting: too complex for given amount of data, sensitive to small changes in the sample (e.g., outliers) • Unrealistic predictions (e.g., proportions >100% or <0%)	Cite with caution, redo data analysis if raw data available	(Kruschke & Liddell, 2018; McElreath, 2018)
Conclusions unwarranted	Accepting the null	p >.05 or 95% CI that overlap with zero interpreted as evidence of no effect	Low power, no formal equivalence testing, but conclude that “X has no effect on Y”	• Replace “no difference” with “we don’t know” • When citing, distinguish between actual results and authors’ claims	(Kruschke & Liddell, 2018; Wagenmakers et al., 2018)
	Over-generalization	Unwarranted generalization from narrow results to sweeping conclusions	Unbalanced designs (many more subjects than stimuli, unrepresentative sample of subjects), likely confounds, conclusions/abstract stated in much broader terms than methods		(Yarkoni, 2022)
	Vague theoretical predictions	No one-to-one correspondence between theories and predictions	Competing hypotheses are weakly probabilistic and compatible with multiple theories, impossible to prove or falsify		(Muthukrishna & Henrich, 2019; Oberauer & Lewandowsky, 2019)

There are valid reasons to withhold the data, such as protecting the participants, and many researchers express reservations about releasing their data for fear of being scooped by other teams. However, the practice of sharing research data is increasingly encouraged by journals and funding agencies and gradually adopted by researchers (Fecher et al., 2015; Houtkoop et al., 2018). The absence of data in a relatively recent publication – or, even worse, authors’ unwillingness to provide data upon request, without a good reason – can be a warning sign because without raw data it is much more difficult to detect fraud or to verify the analyses. For instance, when the editor of *Molecular Biology* T. Miyakawa requested to see the data from 41 articles with suspiciously beautiful results, 21 were immediately withdrawn by the authors and another 19 were subsequently rejected because the provided data were insufficient or inconsistent with the reported results (Miyakawa, 2020). Fortunately, dark data magic leaves traces. If the data are manipulated to fit the hypothesis or reach the significance threshold (falsified), outliers tend to be removed, reducing overall variation, and the reported effects may be unnaturally large (Bordewijk et al., 2021; Hartgerink et al., 2019). There have also been attempts to detect fraud by examining distributions of significant digits in the reported regression coefficients (Diekmann, 2007) and other summary statistics (Hartgerink et al., 2019; Hüllemann et al., 2017), which should follow Benford’s logarithmic law in many natural measurements (Hill, 1995). However, this method produces a lot of false alarms, and Benford’s law is only applicable when the analyzed values range over several orders of magnitude and are not bounded. If data are generated by a computer algorithm using realistic distributions, there will not be any obvious digit preferences or anomalies in data distribution, but subtle signs of fabrication may still be present – for instance, groups in a randomized study may be implausibly well matched (Al-Marzouki et al., 2005; Bolland et al., 2024; Carlisle, 2017), or the fraudsters may omit to recreate the complex pattern of correlations normally found in real data (Bordewijk et al., 2021).

It is widely recognized that we need simple and reliable tools for detecting fabricated or falsified data. Several R packages and other tools have been developed for performing simple statistical integrity checks, detecting plagiarism, etc. (Bolland et al., 2025; Bordewijk et al., 2021; Heathers, 2025a). So far, automatic checks are mostly limited to detecting simple inconsistencies in reporting and may only work if the text is correctly formatted. More advanced and specialized tools fall within the scope of what might be called “forensic metascience” (Heathers, 2025a). These may be an overkill for regular readers who are not editors or data experts, but anyone can draw a scatterplot of the provided dataset and eyeball it for obvious inconsistencies, which can also be a first step toward verifying the reported data analyses if those also seem problematic.

## There are not enough data

Assuming that the data are genuine, the next major stumbling block is not having enough of it. The term *power* refers to the probability of making the correct binary decision in the context of hypothesis testing (e.g., of detecting a true effect), which can be based on null-hypothesis significance testing (NHST) (Cohen, 1962, 1992) or alternative Bayesian techniques such as Bayes factors or posterior probabilities (Kruschke & Liddell, 2018). An alternative goal may be to estimate an effect with sufficient *precision* instead of accepting or rejecting a hypothesis (Kelley et al., 2003), but the problem is conceptually similar regardless of the exact statistical procedure: there may not be enough data to answer the research question meaningfully.

Underpowered, small-sample studies are usually justified along the lines of “anything is better than nothing” and “we have to start somewhere” (Yarkoni & Westfall, 2017). However, numerous statistical problems are compounded in small samples, including the influence of inappropriate priors and outcome distributions, whereas large datasets are more forgiving of imperfect analytical techniques and less likely to lead to misleading conclusions (Wagenmakers et al., 2018). Low power also compounds the problems of publication bias and inappropriately accepting the null hypothesis (see below). Even if the results are statistically significant but the power is low, the reported estimates are likely to be gross exaggerations of the true effect sizes, which might even go in the opposite direction – simply put, statistical significance does not mean much in underpowered studies (Gelman & Carlin, 2014). These major issues with underpowered studies have been repeatedly raised since the 1960s (Button et al., 2013; Cohen, 1962; Ioannidis, 2005; Kelley et al., 2003; Schmidt, 1996; Sedlmeier & Gigerenzer, 1992; Smaldino & McElreath, 2016), with each new review repeating the warnings and concluding that there is little sign of improvement, at least in behavioral and social sciences. Naturally, many studies simply cannot be conducted with large samples – consider clinical research on rare diseases or costly animal experiments. However, the research questions and study design then have to be adapted to these practical constraints, and if they are not, the results are suspect. In animal research, for instance, we can ask simpler questions with large expected effects, increase sample size through multi-lab collaborations, use informed priors based on expert opinion, or focus on qualitative descriptions and case studies (Farrar et al., 2020, 2023). In neuroimaging, the typical sample size of about 30 is usually inadequate, but the power can be improved by using multivariate techniques that compare whole-brain activation patterns rather than individual voxels (Bossier et al., 2020; Botvinik-Nezer & Wager, 2023; Klapwijk et al., 2021; Marek & Laumann, 2024).

How can a reader know if the study they are looking at is underpowered? Merely checking the reported *N* is not enough: what counts as a small sample depends on the research design, expected effect size, and variability of outcomes. A study may be grossly underpowered even if its sample size is in line with conventions, so reference to similar previous research is not a valid justification. As separate ANOVAs of data aggregated per participant (F1 analysis) and per item (F2 analysis) are giving way to multilevel or mixed models, the very concept of sample size requires rethinking. A common mistake is to equate sample size with the number of tested participants and ignore other clusters such as test items. It is well understood that inference to the population of subjects is invalid if the tested sample is not representative of this population (Henrich et al., 2010). But optimal power in the context of multilevel modeling also requires sampling a sufficient number of units at each level: for instance, 40 subjects × 40 items is better than 200 subjects × 8 items (Brysbaert & Stevens, 2018; Westfall et al., 2014). The contribution of both subject and item sample sizes should also be made explicit by modeling both as random effects (Barr et al., 2013; Yarkoni, 2022). Simple rules of thumb have been proposed for typical design, such as having at least 20 observations per cell in contingency tables (Simmons et al., 2011, p. 1362), or at least 1,600 observations per condition (Brysbaert & Stevens, 2018, p. 1), but no rules can fit all possible designs.

Fortunately, there is a simple tell-tale sign that a published result is based on insufficient data, namely high uncertainty in the reported point estimates – just look for wide error bars (standard errors, confidence intervals, or Bayesian credible intervals). This is not about whether the effect size is large or small in natural or standardized units: the question is how precisely it is estimated. Thus, a group difference with Cohen’s *d* of.1 ±.01 is substantively small but estimated with high precision, whereas a *d* of.8 ±.5 may seem excitingly large, but too imprecise to be taken seriously. While there is an ongoing debate about the importance of hypothesis testing versus effect size estimation (Kruschke & Liddell, 2018; McShane et al., 2019; Meehl, 1978; Schmidt, 1996; Wagenmakers et al., 2018), the gradual transition from NHST to the New Statistics with confidence intervals (Cumming, 2014), and even more so to the Bayesian New Statistics with posterior distributions (Kruschke & Liddell, 2018), has made the issue of insufficient data much more transparent. Whereas a p-value or Bayes factor provide no information about the uncertainty in the estimated effect size, a Bayesian credible interval (either quantile-based or the highest density region of the posterior distribution) provides an intuitive measure of the level of precision achieved by a study, and it is straightforward to calculate for any effect of interest, regardless of model complexity (Kruschke & Liddell, 2018; McElreath, 2018).

The precision of effect size estimates can be used as a quick proxy for power only if the statistical analysis is reasonably appropriate, which is often not the case. For instance, standard errors may be mislabeled as confidence intervals in a plot, relevant random effects omitted, or observations aggregated within clusters, all of which inflate the apparent precision of estimates. If no uncertainty estimates are provided either in the plots or in the text, other signs of insufficient data are small samples at any level at which inference is made to a larger population (e.g., too few individuals, items, tasks, countries, research centers, etc.), intrinsically noisy measurements (e.g., animal behavior), unexpectedly large and exciting results, influential outliers (e.g., single points in scatterplots that appear to anchor the regression line), and statistical analyses that are opaque or excessively complex given the amount of available data. Finally, it is worth checking the sensitivity analyses, if any are reported. There may be too little data relative to the observed effect size if the substantive conclusions vary across several reasonable analytic approaches – for instance, if the effect suddenly disappears after we take the logarithm of a right-skewed outcome or remove a couple of outliers. In short, a properly measured, trustworthy effect should be fairly robust and established with enough precision to answer the research question unambiguously.

## The data are not analyzed properly

Suppose we see no indication that the data have been tampered with, and there seems to be enough of it. Can we believe the authors’ conclusions, then? Not yet: it is time for a closer look at the description of research design and data analysis. Potential problems are too numerous to discuss here in full, and the best way to get better at detecting them is to learn statistics and gain experience by critically reading and reviewing papers, but a few are both widespread and relatively easy to detect.

The first major concern is that the analyses may be too flexible: the data are tortured until they confess something, anything – and if they confess the wrong thing, the question is reframed accordingly. The way this is done is partly field-specific. In medical trials, for instance, the analyst may abuse subgroup analyses, switch outcomes relative to the preregistration plan, or experiment with missing data handling until the desired effect is observed (Grey et al., 2020). In psychology, data dredging (p-hacking) and Hypothesizing After the Results are Known (HARKing) get the most attention. Because most effects are probabilistic rather than deterministic, and because they are measured with some noise, there is always some non-zero probability of detecting an effect where there is none (false positive) or missing a true effect (false negative). The more tests are performed, the more likely we are to make false discoveries. Therefore, procedures like multiple testing and stepwise model selection inflate the rate of false positives beyond the nominal alpha level used in individual significance tests – this is data dredging (Gelman & Loken, 2013, 2014; Ioannidis, 2005; Simmons et al., 2011). This is particularly problematic when the decisions about data collection and analysis, collectively known as researcher degrees of freedom, remain hidden: dozens of tests may be performed, but only the ones that have produced significant results are reported (known as “selective reporting”). Even worse, theoretical predictions may be (re)written after the analysis to fit the results – this is HARKing (Kerr, 1998), which half the researchers admit engaging in (Rubin, 2017). There is a huge literature on why p-hacking and HARKing are so harmful (Gelman & Loken, 2013, 2014; Ioannidis, 2005; Kerr, 1998; Munafò et al., 2017; Nosek et al., 2019; Rubin, 2017). In essence, problems arise when exploratory research is presented as more confirmatory than it really is, inflating false positives – we pretend that the prior plausibility of the hypothesis that fits the findings is higher than it really is.

Nearly all discussions of p-hacking and HARKing recommend preregistration and replication as the ultimate solution (Forstmeier et al., 2017; Gelman & Loken, 2014; Munafò et al., 2017; Nosek et al., 2018, 2019; Rubin, 2017), but there is rather mixed evidence on whether preregistration reduces p-hacking and HARKing in practice (Brodeur et al., 2024; van den Akker et al., 2024). Sometimes preregistration is simply impossible, as in much observational sociological research (Gelman & Loken, 2014), whereas in disciplines such as economics preregistration may do little to curb unreported researcher degrees of freedom because it does not require disclosing a pre-analysis plan (Brodeur et al., 2024). Moreover, many researchers balk at the perceived inconvenience and loss of flexibility associated with formally preregistering a study and its data analysis plan (Nosek et al., 2019). My personal recommendation would be simply to share all analysis scripts and datasets to enable exact replication of the analyses (Nosek et al., 2019; Oberauer & Lewandowsky, 2019; Simmons et al., 2011). Failing to reproduce the analysis described in the text is a surprisingly common problem (Artner et al., 2021; Miłkowski et al., 2018), and in data-heavy fields like computational modeling there are calls for sharing the entire software ecosystem with all software dependencies necessary for the analysis (Botvinik-Nezer & Wager, 2023; Miłkowski et al., 2018). In most fields, however, it should be enough to share R markdown or Jupyter notebooks containing both scripts and their outputs. If these are available, I would not worry too much about whether or not the study was preregistered. Furthermore, preregistration is no guarantee that the analysis was performed and interpreted correctly, so the conclusions of preregistered studies still need to be examined critically, and the preregistered analysis plan has to be compared with the final publication.

Assuming that analysis scripts and/or plans are provided, major smoking guns to watch out for are things like running many more tests than reported in the paper, performing stepwise model selection based on likelihood ratio tests or information criteria, or trying various model structures until one produces a significant effect (Forstmeier et al., 2017; McElreath, 2018). Even if these steps are hidden from the reader, the end result may be revealing in itself – for instance, the authors may present an arbitrary-looking, theoretically far-fetched model with multiple significant covariates and interactions. A classic signature of HARKing is convoluted and far-fetched reasoning in the final paragraph of the Introduction, which twists the literature to predict precisely the effects and complex interactions that are then “confirmed” in Results. There may also be a strange mismatch between the stated hypotheses and experiment design, as if the study was initially designed to test a different question (Kerr, 1998). Going Bayesian is not an antidote to p-hacking and HARKing: it is just as easy to HARK about the observed posterior distributions and to hack with Bayes factors (Kruschke, 2021; Simmons et al., 2011). Anecdotally, a Bayesian analysis may even be employed in a last-ditch bid to salvage the paper if none of the traditional tests produced a significant p-value. Obviously, this is not very useful: a broad posterior distribution may be more informative than a large p-value, but a weak effect is still a weak effect, Bayesian or not.

A particularly glaring form of HARKing is to observe the effect direction, “predict” it theoretically by selectively citing the literature that fits, and then use a one-tailed hypothesis to “confirm” this prediction. Here is a real-life example. In the preferential looking paradigm, a difference in looking times is interpreted as preference for congruency if subjects look longer at the congruent stimuli, or as surprise at a violation of expectations if they look longer at the incongruent stimulus. Not only is a difference in either direction accepted as evidence for the theory, but one-tailed statistical tests are sometimes applied, as if the direction of effect was known in advance. For instance, the reported congruence effect in a study of audiovisual crossmodal correspondences in human infants (Walker et al., 2010) is no longer significant if a two-tailed binomial test is applied instead of a one-tailed test. As a side note, this widely accepted finding consistently failed to replicate in a heroic, yet barely cited, series of follow-up experiments that increased Walker’s original sample size from *N* = 16 to a jaw-dropping total of 376 infants (Lewkowicz & Minar, 2014). While infants may well be sensitive to crossmodal correspondences, methodologically what we have here is a perfect storm with small samples, extremely noisy outcomes, a healthy dose of HARKing, and publication bias, which in combination all but guarantee a high rate of false positives. Another lesson to draw from this example is that the number of citations is a very poor predictor of methodological quality or replicability; in fact, studies that fail to replicate continue to be cited as much as before (Schafmeister, 2021; Serra-Garcia & Gneezy, 2021).

**Fig. 1:**
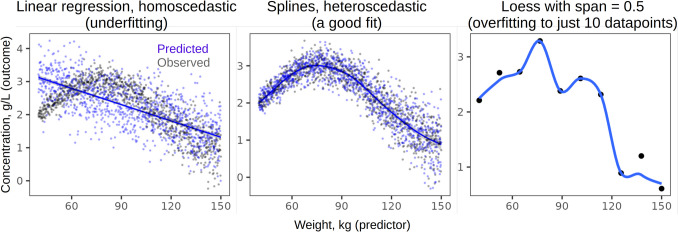
Model fit can be checked by plotting the observations (black points) together with fitted values (blue lines) and model predictions (blue points). Linear regression with a fixed standard deviation in this case underfits the data, whereas a Generalized Additive Model with spline smoothing captures the underlying sinusoidal trend perfectly. If only 10 datapoints are sampled from the same generative process, LOESS smoothing with a fixed span of 0.5 overfits the data. Inference from severely underfit or overfit models is highly suspect. Note that Pearson’s correlation assumes a linear relationship between variables, so it would be meaningless to report Pearson’s *r* between these two variables

Another major pitfall in data analysis is using unrealistic or misspecified models. Obviously, there are countless ways to bungle up the statistics – many more than can be discussed here – but many major mistakes that slip past the peer review appear to fall into this category. Drastic violations of model assumptions lead to unrealistic predictions or invalid inference, and yet there is a pervasive tendency to treat all data as Gaussian, linear, homoscedastic, and independent. Some violations are not likely to change the results of hypothesis testing: for instance, Gaussian models provide robust inference even when the errors are not at all normally distributed (Knief & Forstmeier, 2021). Still, effect sizes will be affected by poor model fit, not to mention that it is meaningless to predict impossible values such as success rates over 100% when fitting a Gaussian model to proportions. Even more consequential mistakes include modeling strongly nonlinear effects as linear (Fig. 1), ignoring heteroscedasticity (dependence of data variance on a predictor), or failing to model autocorrelation in temporal data (Knief & Forstmeier, 2021). All these problems are compounded by working with small samples (see above on low power), although large datasets are also vulnerable to overfitting and data leakage between training and testing sets (Yarkoni & Westfall, 2017). While potential problems with model fit are legion, many can be detected by simply plotting model predictions over raw data (Fig. 1). Limited space in the main text may preclude showing all these plots, but they are easy to include in supplements, especially if the scripts are shared as notebooks. Readers can also make these plots themselves from the datasets – provided these datasets are shared – and compare them to the plots in the publication (Kruschke, 2021).

## The conclusions are not justified

**Fig. 2:**
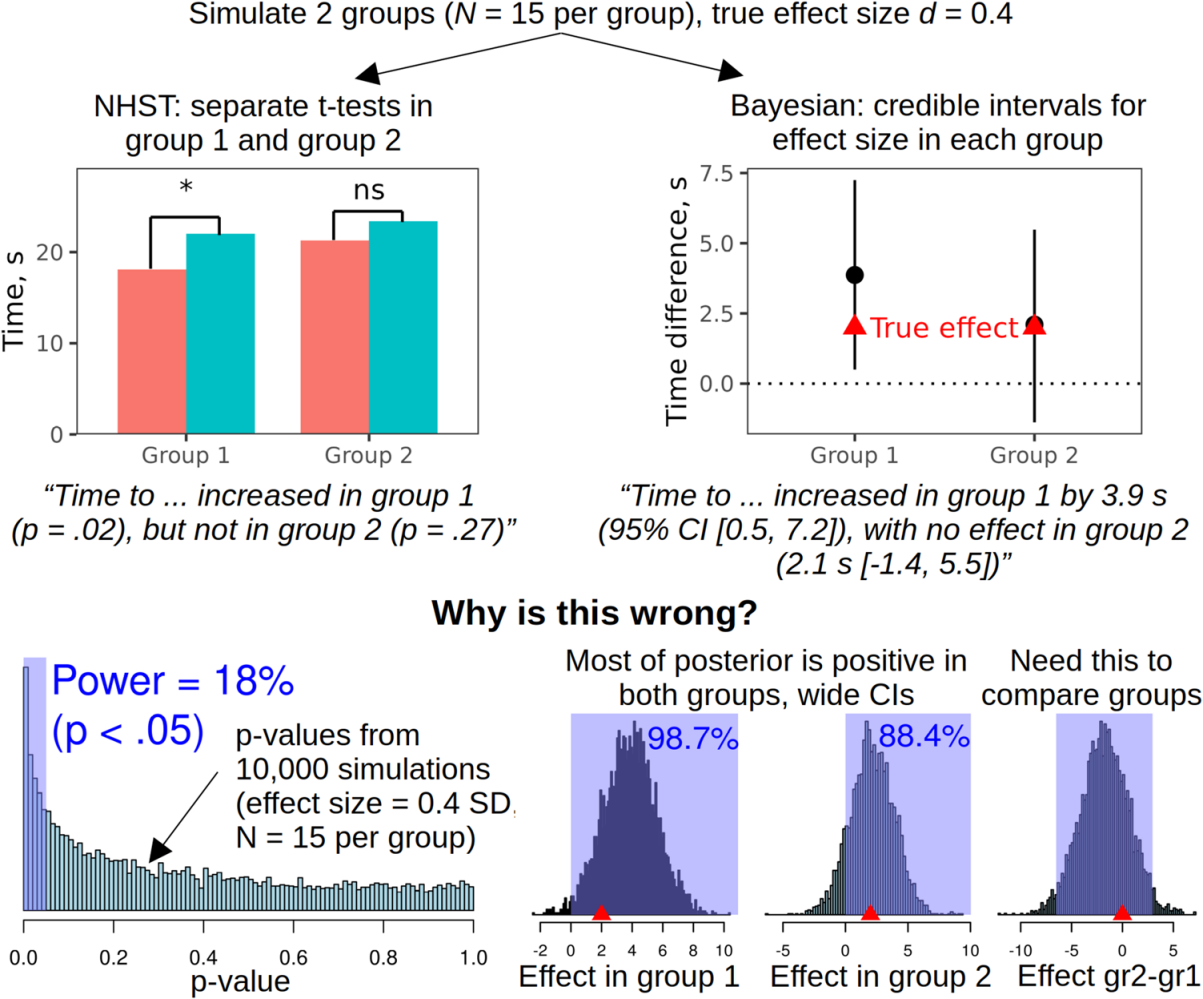
A simulation demonstrating some perils of inappropriate hypothesis testing in combination with insufficient data. It is tempting to interpret the non-significant p-value or the 95% credible interval (CI) that includes zero as evidence of no effect in group 2, but these are not valid ways to prove the null hypothesis (Bayes factors or equivalence testing could be used instead). Note also that the power is just 18% and the CIs are very wide, suggesting that there is simply too little data to estimate the effects in both groups with any certainty. If we specifically want to compare the effect in groups 1 and 2, we need to test for a treatment x group interaction or obtain the posterior distribution of the difference in the effect between groups, as shown above. NHST = null-hypothesis significance testing. Code: https://osf.io/n7r2y/

Let us assume that the data are genuine and competently analyzed. There are still several pitfalls on the way from the output of statistical models to the conclusions. A very common mistake is to interpret a *p* >.05 or a confidence interval that overlaps with zero as evidence of no effect. A theoretically meaningful null hypothesis can indeed be accepted using Bayes factors (Dienes, 2014; Wagenmakers et al., 2018) or frequentist equivalence testing (Lakens et al., 2018). In a Bayesian setting, for instance, we can be 95% certain that the effect is too small to be of practical significance if 95% of posterior distribution is contained within the Region of Practical Equivalence or ROPE (Kruschke & Liddell, 2018). A large p-value, in contrast, does not prove the absence of an effect either logically (Goodman, 2008) or even as a rough approximation as there may simply be too little data. Likewise, a frequentist confidence interval or Bayesian credible interval may overlap with zero simply because the precision of estimates is low (Kruschke & Liddell, 2018). A typical *faux pas* is to observe two substantively similar effects in two treatment groups and to draw a strict distinction in the interpretation based on which side of *p* =.05 they land on (Fig. 2).

This is particularly misleading when the study is underpowered because, by definition, low power means a high probability of failing to detect true effects. Because most studies in psychology are indeed underpowered (see above), and because inappropriate acceptance of the null hypothesis is overwhelmingly common in the literature, readers must exercise great caution and mentally substitute “we don’t know” for a lot of published claims of “no difference.” The opposite mistake is to place too much faith in small p-values or narrow confidence intervals in high-powered studies, equating statistical significance with practical importance (Goodman, 2008; Schmidt, 1996). Every possible effect becomes significant at some point as more data are collected, but the effect sizes must be interpreted in practical terms and contextualized: for example, a highly significant risk reduction of 15% becomes less exciting if the baseline risk is just one in a million. Both problems can be addressed by paying more attention to effect sizes and their uncertainty expressed in natural, meaningful units (e.g., incidence rates rather than log-odds).

The second fallacy in interpreting empirical data is believing too implicitly in the conclusions of systematic reviews and meta-analyses. The statistical techniques of meta-analysis were developed to pool all available evidence and overcome the limitations of individual studies, notably their insufficient power (Borenstein et al., 2021; Cumming, 2014; Schmidt, 1996). A crucial prerequisite is that the sampled studies should be representative of all research conducted (Borenstein et al., 2021; Schmidt, 1996). Unfortunately, there are two selection biases that distort the result of meta-analyses. The first is the publication bias or file-drawer effect: large and statistically significant effects get published, whereas null results end up in a drawer (Button et al., 2013; Carter et al., 2019; Franco et al., 2014; Iyengar & Greenhouse, 1988; Rosenthal, 1979). As a result, positive findings are reported in nearly 100% of regular publications, but in less than half of registered reports, which are accepted for publication before data collection takes place (Scheel et al., 2021). Just as a biased sample in a single experiment leads to invalid population inference, a biased sample of empirical evidence in a meta-analysis produces excessively optimistic estimates of the true effect because unrealistically large effects are more likely to be observed by chance in small samples (Etz & Vandekerckhove, 2016; Forstmeier et al., 2017; Rothstein et al., 2005; Smaldino & McElreath, 2016). Thus, publication bias is similar to p-hacking – both only show what worked and bury the rest (Simonsohn et al., 2014). A variety of statistical tools have been proposed for detecting and correcting for publication bias (Adler et al., 2023; Borenstein et al., 2021; Iyengar & Greenhouse, 1988; Rosenthal, 1979), but it is difficult to account for it fully, particularly when individual studies are small (Carter et al., 2019; Rothstein et al., 2005).

**Fig. 3:**
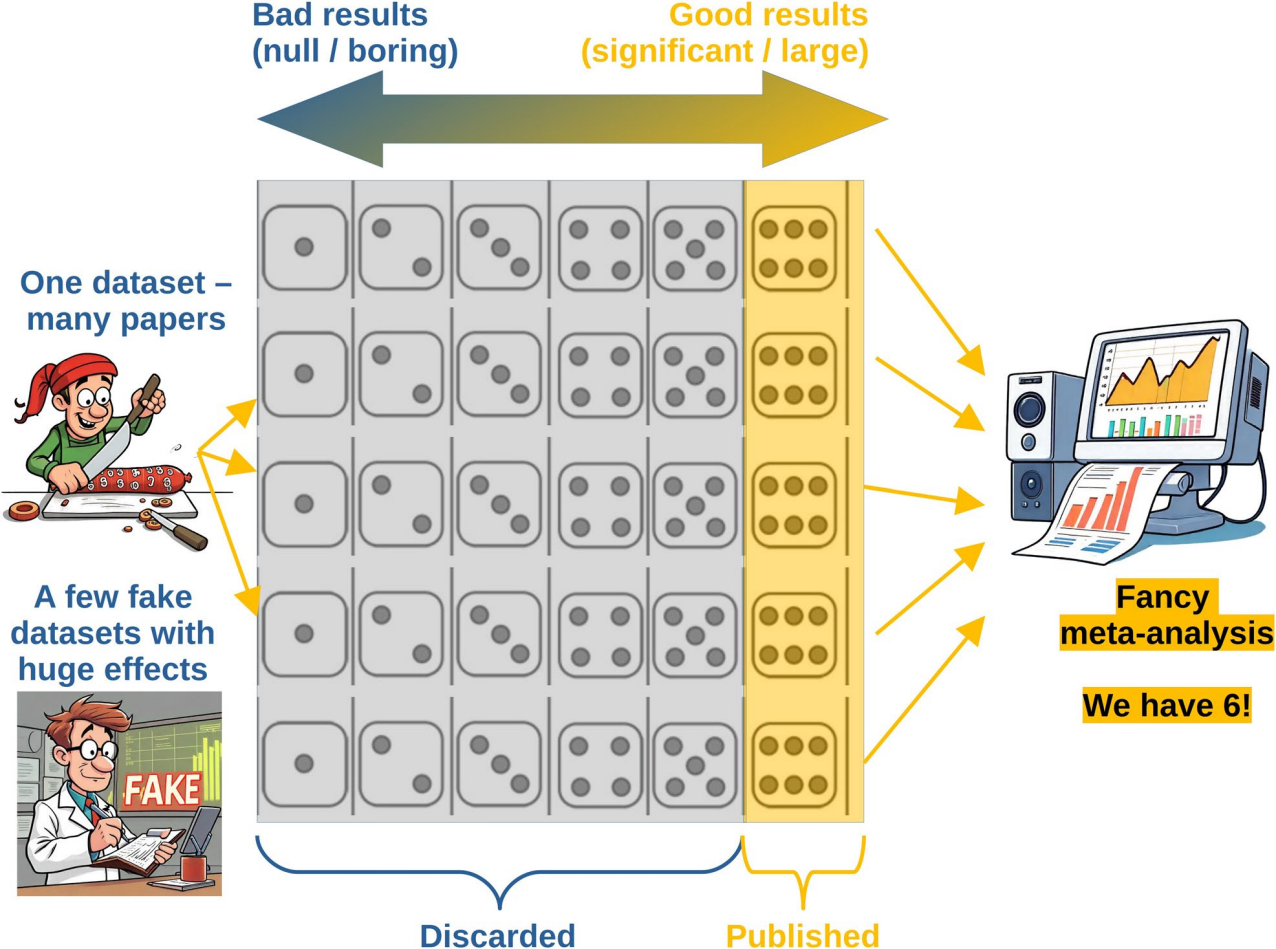
Meta-analyses can reach overly optimistic conclusions if studies with significant and large effects are more likely to be published. Salami slicing a single dataset into multiple publications and including fraudulent data can further skew the results. Source of images: https://clipart-library.com

While publication bias gets most of the attention, there are other sources of bias in meta-analyses. For instance, “salami slicing” the results of a single study into several small publications – a shady but very common practice also known as selective publishing (Nagy et al., 2024) – can skew the results of meta-analyses because multiple publications based on the same dataset are treated as independent datapoints (Antonakis, 2017; Bolland et al., 2025; Saiz et al., 2018; Williams & Roberts, 2016). A meta-analysis can also be “poisoned” by including one or two fraudulent studies showing improbably large effects (Bolland et al., 2022). For example, several meta-analyses have concluded that vitamin D reduces the risk of bone fractures, but its beneficial effect disappears after excluding two suspicious studies with the largest effect sizes, one of which is now formally retracted (Bolland et al., 2025, p. 274). Thus, meta-analyses are only as good as the evidence they are based on: as in the rest of statistics, it’s garbage in, garbage out (Fig. 3). Obviously, a reader cannot verify all the studies entered into a meta-analysis – the message is simply to treat meta-analyses as data points, more convincing than single small studies but by no means infallible.

The third major category of unjustified conclusions is exaggeration or over-generalization of trivial or uninformative results. I already touched upon one aspect of it when discussing low statistical power, namely the need to consider the number of stimuli, tasks, and other grouping factors in addition to subjects as components of the sample size (Gelman & Brown, 2024; Yarkoni, 2022). The problem is not narrowly statistical, however; it is tempting to make the findings sound more general and impressive than they really are. This logical leap of faith occurs somewhere between the Results and Conclusions of a paper, and readers can detect it by asking themselves whether the conclusions can be restated using narrower terms. For instance, if we studied the healing of minor bruising with suction cups, would the results necessarily generalize to other types of injuries (Gelman & Brown, 2024)? If crossing out the letter *e* slightly decreases response accuracy on a subsequent Stroop task, is this good evidence of ego depletion (Yarkoni, 2022)?

Another key question to ask before accepting the conclusions at face value is whether we are making a logical leap from correlation to causation, and whether there might be hidden confounds or alternative explanations. For instance, amusement park rides are perceived as more exciting if their names are hard to pronounce (Song & Schwarz, 2009), or are they? Bahník and Vranka failed to replicate this effect in seven studies and concluded that pronounceability was confounded with word length, which was far from obvious because there were only three pairs of stimuli in the original study (Bahník & Vranka, 2017). This is an example of generalizing over unmodeled sources of variance (in this case, having too few and insufficiently diverse stimuli), which can be addressed by testing a greater variety of stimuli, manipulations, linguistic and cultural groups of participants, etc. (Henrich et al., 2010). Or consider this: are words with inward articulation, such as MADIKO, preferred over words with outward articulation, such as KADIMO? From the embodied emotion perspective, inward-moving consonants should be associated with ingestion, and outward-moving with spitting out food (Topolinski et al., 2014), but no link with disgust is reported, so perhaps the effect is confounded with a general preference for articulating in the front of the mouth rather than in the back (Maschmann et al., 2020), articulation fluency (Ingendahl & Vogel, 2022), temporal organization of planning articulatory gestures (MacDonald & Weiss, 2022), or some other as-yet mysterious factor (Ingendahl et al., 2022; Topolinski et al., 2024).

Such debates are ubiquitous in psychology and related disciplines, and they are not solely due to paucity of evidence, but also to non-specific theoretical predictions and lack of an overarching framework. Broad theories like embodied emotion are compatible with almost any empirical results and can therefore be neither proven nor falsified empirically. When multiple theories make the same vague and weakly probabilistic predictions – that X is affected by Y, not that X is going to be 23% higher if Y is doubled – neither positive nor negative results help to adjudicate between these theories (Muthukrishna & Henrich, 2019; Oberauer & Lewandowsky, 2019). As a result, theories proliferate and are treated like toothbrushes – “no self-respecting person wants to use anyone else’s” (Mischel, 2008). Instead of being proven or falsified, they come into fashion and then fade away as people lose interest, which is incompatible with cumulative science (Muthukrishna & Henrich, 2019; Schmidt, 1996). The practical message for a reader is not to be too quick to accept every grand conclusion or to (re)interpret every result in the light of their own favorite theoretical paradigm, but to keep an open mind and stay closer to the actual facts. If you take notes, like me, write down what the authors did and what they found in simple terms – stimuli and manipulations, subjects, effects sizes, uncertainty – and do not merely copy the “big picture” from the Abstract and Conclusions. Even if the data are bona fide and the analyses impeccable, the conclusions may still be unwarranted or exaggerated.

## What to do with papers we don’t trust?

The objective of this review is to alert readers of scientific papers to some common problems which cast doubt on many printed claims that are based on quantitative – and seemingly solid and objective – evidence. The question remains, however, what to do once a serious fault in the data, analysis, or theoretical conclusions of a publication is discovered. By finding a shaky claim, we have already learned something personally valuable; for instance, it would be pointless to plan our future research based on a finding that is not likely to replicate. For cases of suspected fraud or misconduct, readers should contact the authors and then the journal, post concerns on websites such as PubPeer (https://blog.pubpeer.com/), or contact the Committee on Publication Ethics (https://publicationethics.org/). By all accounts, ensuring the retraction of a fraudulent paper is likely to be a protracted and unrewarding process (Bolland et al., 2025; Saiz et al., 2018). An unintentional critical mistake in the analysis may warrant retraction or at least a corrigendum. However, most weaknesses described above (e.g., low power, talking about null results as if they proved the absence of an effect, or over-generalization of the results) are more or less standard practice, and they are likely to remain so in the foreseeable future. Furthermore, many problems are likely to be honest mistakes rather than cases of scientific misconduct, with a large gray zone of Questionable Research Practices like p-hacking in between these extremes (Nagy et al., 2024). The point is not to apportion blame but to decide whether and how to cite dubious claims.

One option is simply to ignore studies that appear to be of poor quality or make unwarranted claims, omitting them from literature review. However, studies cannot be arbitrarily removed from a meta-analysis or a systematic review (Borenstein et al., 2021). Even in an empirical paper, reviewers are likely to question the failure to mention clearly relevant previous research – particularly if it is written by the reviewers themselves. Furthermore, there is a slippery slope from omitting poor-quality papers to cherry-picking the literature to cite. A less drastic course of action is to cite the questionable paper or claim, but to explain why it may be incorrect. Unfortunately, this option is also wide open to abuse as almost any inconvenient result can be explained away: the sample is not large enough or diverse enough, possible confounds are not accounted for, etc. Following Yarkoni (2022), I propose to make a clear distinction between citing the claims and citing the evidence. To return to the earlier example of articulation dynamics, instead of writing “words that are difficult to pronounce are perceived as risky (Song & Schwarz, 2009),” we can separate the data from its interpretation: “Song and Schwarz (2009) report that 35 American students rated written words *Chunta, Ohanzee*, and *Tihkoosue* as 20% riskier than *Vaiveahtoishi, Tsiischili*, and *Heammawihio* when these were presented as names of amusement park rides, which the authors interpret as evidence that low processing fluency translates into greater perceived risk.”

Verifying published claims and clarifying what the actual evidence is, rather than merely echoing the authors’ conclusions, can go a long way toward curbing further spreading of questionable papers and unwarranted claims. However, it places a heavy burden on individual researchers, who may lack the time, analytical skills, or motivation to check the validity of the datasets, study design, scripts for statistical analysis, and claims in the primary research they read and cite. A close scrutiny may be justified in the case of key findings that determine public policy or inform future research. Realistically, however, much of the collective responsibility will still fall upon the authors of meta-analyses and systematic reviews. If data sharing is enforced, it should soon be possible to meta-analyze raw observations from multiple studies instead of summary statistics, bypassing concerns about the original analyses. The included studies will also need to undergo comprehensive checks of their methodological integrity, which requires expert knowledge of the study domain and blurs the line between quantitative meta-analyses and more traditional narrative systematic reviews (Borenstein et al., 2021). Studies may even be weighted by overall quality rather than by sample size alone, followed by sensitivity analyses to test to what extent the conclusions are swayed by suspicious or poor-quality studies (Bolland et al., 2025). In turn, theoretical reviews should become more discriminating in the evidence they cite, disentangling chains of references back to the original empirical data and verifying it, re-analyzing the raw data when available, or calling for large replication studies when not available. This is a huge task, but a necessary one if we are to kill zombie hypotheses and ensure cumulative progress (Forstmeier et al., 2017). In the meantime, every researcher can avoid wasting their time on an unproductive avenue, as well as contribute toward making psychological science as a whole more replicable and trustworthy, by becoming better at critically evaluating published evidence. This is a particularly essential skill to develop in the next generation of researchers, calling for a greater emphasis on critical reading as part of professional training in research design and data analysis. This will produce stronger researchers and in the long run enhance the legitimacy of science itself.

## Data Availability

The data for reproducing the figures is available at https://osf.io/n7r2y/.

## Code availability

The code for reproducing the figures is available at https://osf.io/n7r2y/.
